# Therapeutic Notes

**Published:** 1892-04

**Authors:** 


### Progress in Medical Science.

### THERAPEUTIC NOTES.

For forming bubo from whatever cause (*Medical Fortnightly*), the application of a wad of absorbent cotton, soaked in fluid extract of phytolacca deccandra, kept moist by a covering of gutta percha tissue, has been found to have marked controlling effect. The use of this remedy was suggested by its usually prompt dissipation of caked breast, when applied in a similar way.

### INTERNAL ANTISEPTICS.

Salol is the best of the internal antiseptics, (*Dujardin-Beaumetz,*) because it is always well borne by the digestive tract; it is but slightly soluble, and is decomposed into carbolic and salicylic acid only in an alkaline medium, *i. e.*, in the intestine. Iodoform and naphthol, which are always toxic and irritant, are muchinf erior to it, for it is but slightly toxic. Almost equally valuable is the salicylate of bismuth, which acts on both the stomach and intestine.

℞ Salol. ............................
Bismuth salicyl ...................
Sod. bicarb ...................... aa ʒ iiss.
Div. in caps. xxx.

### ENTERIC FEVER TREATED BY LIQUOR SODA CHLORINATE.

The antiseptic treatment of typhoid fever has been so highly lauded by some physicians, and has so often proved useless, that the latest suppliant for popular favor is worth mentioning,—namely, chlorinated soda. Pearson, of Cape Colony, writes to the *Lancet*, of December 5, 1891, that he has found the following method extraordinarily successful in a very large number of cases. He prescribes from fifteen to twenty minims every three or four hours of a solution made as follows, and gives no other medicine whatever, maintaining a milk diet : The solution is made by dissolving one and a half pounds of carbonate of sodium in twenty-four ounces of water, and by triturating thoroughly a pound of chloride of lime in 120 ounces of water. These liquids are now filtered and mixed together, and then again filtered. The solution should be perfectly clear and free from traces of lime, and should be kept in a cool place. This treatment Pearson continues until the temperature has remained normal for two days.—*Therapeutic Gazette.*

---

### TREATMENT OF SHOCK.

CHAUVEL recommends the following treatment of shock, according to *La Médecine Hypodermique* for January, 1892: For the purpose of reëstablishing the circulation, the patient is put in an absolutely horizontal position and massaged. He is also given alcoholic frictions and subcutaneous injections of ether. For the purpose of maintaining the bodily temperature, the air of the room is well heated, and the patient is surrounded by hot bottles, or placed in a bath of the temperature of 105° or 110°.

Internally, Chauvel administers alcoholic stimulants, such as rum or brandy, in the dose of from one to two ounces. He does not employ sinapisms or other inconvenient measures. When reaction has been obtained, the stimulants are combined with opium for the production of sleep. Chauvel mentions the intravenous injection of ammonia, as has been used by Penfold and Tibbis. In cases where shock is prolonged, strychnine, digitalis, and belladonna are to be employed, and electricity may be of great service.—*Therapeutic Gazette.*

---

### APPLICATION FOR RHEUMATISM.

HEBBING is said to employ the following solution locally in the treatment of rheumatism:

℞—Salol ....................................

Sulphuric ether, aa...................... ʒi;

Collodion................................ ℥i.

To be applied around the joints which are affected by acute rheumatism.—*L' Union Médicale.*

---

### INTERNAL USES OF CHLOROFORM.

IN AN article, entitled The Medicinal Employment of Anesthetics, in *L' Union Médicale*, No. 4, 1892, after calling attention to the fact that chloroform-water is a very valuable remedy in cases of gastralgia, with dilatation of the stomach, in the dose of a small teaspoonful every half hour or hour, attention is called to its value in the treatment of nervous vomiting and of the vomiting of pregnancy. Attention is also called to the fact that Bianchi has found that an aqueous solution of chloroform, in the strength of two per cent., is a useful liquid for the purpose of lavage. It is an analgesic and a moderator of reflex irritability.

Dujardin-Beaumetz has also found that in cases of lithiasis, suffering from gastric pain, chloroform in the following formula is very serviceable :

| | |
|---|---|
| ℞—Saturated chloroform-water................ | ℥ii ; |
| Syrup of orange.......................... | ℥i ; |
| Simple syrup ........................... | ℥i. |

Sig.—A dessertspoonful every fifteen minutes until the pain is relieved.

It has also been found that chloroform is equally efficacious with ether for the removal of gall-stones occurring in gouty persons.

Ulbrecht recommends that chloroform, in the proportion of one to ten, shall be used for the purpose of stopping hemorrhage after extraction of teeth.

The drug is also useful in cases of parasitic painful growths of the mouth, such as the various forms of stomatitis.

Employed in false croup, chloroform, in the proportion of one to ten drops in an ounce of water, to which is added a little glycerin, is very useful when given internally, in the dose of a teaspoonful every half hour. If there is dyspnea through the day, a teaspoonful may be given every two hours and at night every hour.

A very useful employment of chloroform is in cholera infantum in the following prescription:

| | |
|---|---|
| ℞—Syrup of ginger................. | ℥i ; |
| Chloroform..................... | gtt. iii. to iv ; |
| Tincture of opium............... | gtt. x to xv. |

Sig.—A teaspoonful every hour.

In ulcer of the stomach, small doses of chloroform given internally will often remove the pain and stop vomiting, and chloroform-water has also been found useful as a gargle in septic sore throat.

Steep has treated eighteen cases of typhoid fever by the internal administration of chloroform, in the dose of fifteen minims in ten ounces of water, given three times a day during the attack ; at the same time quinine is employed. It is stated that this medication causes a rapid decrease in the fever, and that no disagreeable secondary effects are observed.

Roger has used three to six drops of chloroform, in syrup or mucilage, in the treatment of whooping-cough, with very good results, and in some instances the nervous excitability of delirium tremens can be much quieted by its internal administration. The following prescription is said to have been authorized by Da Costa:

℞—Chloroform........................... ʒi;

Compound tincture of cardamom.......... ℥i;

Syrup of acacia........................ ʒi;

Simple elixir.......................... ℥ii.

Sig.—A teaspoonful every half hour until relief is obtained.

Miller and Griggs are said to have reached very good results in the albuminuria and anasarca of pregnancy by giving chloroform, in twelve- to twenty-drop doses in sweetened water, causing thereby a diminution in the albuminuria and the disappearance of the anasarca. This treatment is also said to have prevented the development of eclampsia.

In the treatment of tape-worm, Kaiser has used the following mixture:

℞—Croton oil........................... m i;
Chloroform............................. m ii;
Castor oil............................. ℥i.

Professional opinion seems to be considerably against the employment of chloroform hypodermically, as after injection of large doses in the lower animals it has been found to create grave renal lesions with degeneration of the muscular fibres of the heart. In man, when it has been employed in the treatment of neuralgia in this manner, it has produced in a number of cases severe pain and profound alterations in the cellular tissues.

In other cases, however, very good results have followed the treatment of neuralgia by injection of from five to ten minims of chloroform into the part affected, although marked edema and swelling of the part has followed the injection.—*Therapeutic Gazette.*

---

### INJECTIONS FOR CHRONIC CYSTITIS.

Ultzmann recommends the following prescriptions in the treatment of this troublesome condition:

Crystallized carbolic acid............. gr. xv;
Distilled water........................ ℥iiiss.

Dissolve, and mix with equal parts of hot water at the moment that the liquid is to be injected.

Or the following:

Boric acid............................. ℥ss;
Glycerin............................... ℥i;
Distilled water........................ ℥x.

Make a solution, and mix with equal parts of warm water at the moment of employment.

Either one of these solutions when warm may be used for washing out the bladder in cases of chronic cystitis. When the vesical secretion is catarrhal and has a bad odor, the following injection is useful :

℞—Nitrite of amyl........................ gtt. v ;
Distilled water.......................... ℥iv.

Mix, and add a tablespoonful of this solution in the proper quantity of water for a vesical injection.—*L'Union Médicale*, No. 12, 1892.

